# Involvement of cognitive abilities in the occurrence of fractures in fallers aged 55 years or older: a cross-sectional study

**DOI:** 10.1007/s40520-024-02830-7

**Published:** 2024-08-30

**Authors:** Elpidio Attoh-Mensah, Kristell Pothier, Gilles Loggia, Remy Morello, Chantal Chavoix, Christian Marcelli

**Affiliations:** 1 Normandie Univ, UNICAEN, INSERM, COMETE, CYCERON, Caen, 14000 France; 2 Department of Psychology, PAVeA Laboratory (UR 2114), University of Tours, Tours, 37000 France; 3 Department of Geriatrics, Normandie Univ, UNICAEN, CHU de Caen, Caen, 14000 France; 4 Department of Statistics and Clinical Research, Normandie Univ, UNICAEN, CHU de Caen, Caen, 14000 France; 5 Department of Rheumatology, Normandie Univ, CHU de Caen, Caen, 14000 France; 6 Permanent address: Univ. Limoges, HAVAE, 123 Avenue Albert Thomas, UR, 20217, F-87000 Limoges, Limoges France

**Keywords:** Fracture risk factors, Fall-related fracture, Bone fragility, Cognition, Executive function

## Abstract

**Background:**

Both bone fragility and poor cognitive functions are known to contribute to fracture occurrence, but it remains unclear whether their contribution is independent of each other and which cognitive dysfunctions are most involved. This study aimed to clarify the involvement of various cognitive abilities in fall-related fractures among community-dwelling fallers aged 55 and over, and to determine whether poor cognitive abilities is a risk factor independent of bone fragility.

**Methods:**

In a cross-sectional study, we collected sociodemographic and medical data, including bone mineral density (BMD), and performed cognitive and mobility assessments in 189 individuals with a history of fall in the previous year.

**Results:**

Fallers with a fracture had poorer cognitive and mobility performance than non-injured fallers. Multivariate regressions revealed that cognition, BMD and other risk factors were independently associated with fracture among all participants (OR = 1.04, 95% CI = 1.01–1.08, *p* = 0.034 for completion time on part A of the Trail Making Test [TMT-A], and OR = 0.53, 95% CI = 0.33–0.84, *p* < 0.001 for BMD), particularly in women (OR = 0.77, 95% CI = 0.60–0.98, *p* = 0.039 for backward digit span score, and OR = 0.43, 95% CI = 0.25–0.75, *p* = 0.001 for BMD).

**Conclusion:**

Thus, poor cognition, especially poor processing speed and working memory, is associated with an increased risk of fracture in fallers, particularly in women, regardless of BMD or other fracture risk factors. Hence, an in-depth cognitive evaluation should enhance the detection of fallers at risk of fracture, particularly in the absence of signs of osteoporosis, and thus ensure the best possible prevention.

**Supplementary Information:**

The online version contains supplementary material available at 10.1007/s40520-024-02830-7.

## Introduction

Fall-related fractures are a major public health issue in older adults due to the number of people affected and serious related consequences. Each year, more than 30% of people aged 65 or over fall at least once, with many of these falls resulting in serious injury, especially fractures (4% of falls) [1, 2]. The incidence of fall-related fractures mainly increases from 50 years of age, with a greater incidence in women than in men aged 50–59 years (80.5% vs. 40.5%, respectively) [3, 4]. This high occurrence in middle-aged and older adults leads to substantial clinical and economic burdens to society through an increase in the rate of hospitalization, admission to emergency care and mortality or harmful consequences for people’s psyche and social life [2]. Therefore, preventing falls and related fractures in older adults has become a worldwide priority [2].

Osteoporosis has been identified as one of the leading risk factors for fractures [5, 6]. This systemic skeletal disease characterized by low bone mass, as determined by bone mineral density (BMD) or microarchitectural deterioration of bone tissues that can be non-invasively assessed by trabecular bone score (TBS), leads to increased bone fragility and fracture risk. The global aging of the population has significantly increased osteoporotic fractures, particularly in Western societies, where the lifetime risk is particularly high (40–50% in men and 13–22% in women) [7, 8]. Notably, bone fragility inherent to osteoporosis is not the only significant risk factor for fractures, as an important proportion of such fractures often occur in people with a normal BMD [5, 9]. One could thus assume that non-osteoporotic risk factors significantly contribute to the occurrence of fall-related fractures. Henceforth, better knowledge and accurate management of these other risk factors are crucial to preventing fracture [9]. These other risk factors mainly include gait disorders, polymedication, comorbidities, alcohol consumption, and cognitive impairment [1, 2].

Cognitive impairment has recently received particular attention because of its well-known association with falls. Indeed, it is now clearly established that there is a close link between cognition and walking and that poor cognitive abilities is a risk factor for falls and fractures [10]. Nevitt and colleagues’ study [11] showed for the first time that poor performance on an executive task was associated with an increased risk of fall-related fractures over a one-year period. Several prospective studies with follow-up periods up to 8 years have further confirmed this involvement of cognition, especially that of global cognition and executive function, in fractures [12–15]. In a systematic review of osteoporotic fracture prediction models, only one of 68 studies considered cognitive impairment, and more precisely impairment in global efficiency, revealing it to be a risk factor [16]. In a recent study we examined cognitive and anthropometric profiles in older women with post-fall limb fractures. Principal component analysis (PCA) identified four profiles, including one with normal BMD and low-medium cognitive performance and another with low BMD and high cognitive performance [17]. However, further research is warranted to elucidate how cognition, bone fragility, and other risk factors interact to increase susceptibility to fractures.

The aim of this study was thus to determine whether poor cognitive abilities is a risk factor for fractures independent of bone fragility in community-dwelling fallers aged 55 years and older. The involvement of specific cognitive dysfunction, identified by the assessment of various cognitive domains, and other risk factors were also investigated.

## Materials and methods

### Participants

Community-dwelling older adults aged 55 and over from a clinical research program at Caen University Hospital (France) were included. The age of 55 years was chosen as osteoporosis-related fracture become increasingly common from that age, especially in women [18]; besides, it is also an age when some cognitive abilities begin to decline.

Participants experienced one or more falls, with or without fracture, in the year before the study. Fallers with fractures were those with upper or lower limb fractures admitted to the orthopedic and emergency departments. Fallers without fractures were mostly recruited at the same hospital by questioning patients in consultation waiting rooms. In particular, they were asked whether they had fallen in the previous 6 months, whether it required medical care and if it did not, whether they would agree to participate in a research study on falls. We chose to include one control (faller without fracture) for every two cases (faller with fracture), a ratio quite acceptable for case-controls studies [19] and supported by previous research [20, 21]. Both fallers with and without fractures were excluded if they had fallen ≤ 2 months or ≥ 6 months ago, were unable to walk for 15 m without help, had pathologies affecting balance, drank > 21 units (for men) or > 14 units (for women) of alcohol per week, had severe depression (Montgomery–Åsberg Depression Rating Scale (MADRS) score > 30) or impaired vision (corrected acuity < 6/10). The lower Normandy Ethics Committee approved the present study (no. 2011A00556-35; clinical trial registration number: NCT02292316__Registration Date November 13, 2014), and each participant provided written informed consent.

### Outcome measurements

Soon after inclusion, participants in both groups underwent several examinations, all carried out on the same afternoon.

#### Bone mineral density (BMD)

BMD was measured at the total hip and femoral neck by DXA using a Hologic 4500 A device, and the lowest T score of the two measured sites was used in the results. Using the World Health Organization criteria, participants were then classified as having a normal BMD (T score ≥ -1.0), an osteopenic BMD (T score between − 1.0 and − 2.5), or an osteoporotic BMD (T score ≤ -2.5 SD).

#### Cognitive and gait measurements

Cognitive and gait evaluations were performed by trained neuropsychologists who were blinded to the participants’ medical records.

*Global cognitive efficiency* was assessed with the Mini Mental State Examination (MMSE) [22] and Montreal Cognitive Assessment (MoCA) [23], two brief 30-point tests, with the latter being more sensitive in detecting mild cognitive impairment.

*Processing speed* was evaluated with three tests: part A of the Trail Making Test (TMT-A) [24] in which the participants must connect numbers randomly displayed on an A4 sheet of paper in ascending order, a copy of the figure of Rey. [25], and the Zazzo’s cancellation task [26] which consists of crossing out 40 target stimuli among 280 distractors. The variables retained were the completion times for the three tests.

*Memory* was assessed as follows: *verbal short-term memory* using the forward digit span test [27], and *visual long-term memory* using the reproduction from memory test of the Rey figure 3 min after copying, which is based on a 36-point scoring system.

In addition, working memory was assessed by two well-known WAIS subtests, the backward digit span test and the letter-digit sequence test [27], where the scores are based on the longest series correctly recalled.

Several *executive functions* were evaluated. *Mental flexibility* was assessed with part B of the TMT (TMT-B) [24], in which the subject must alternately connect numbers and letters in ascending and alphabetical order, respectively. The difference between TMT-B and TMT-A completion times (“TMT B-A”) was used as a relatively pure indicator of executive control abilities [28]. *Verbal fluency* was measured with the Isaacs Set Test (IST) [29], which requires the production of as many words as possible in 4 semantic categories in a given time (15 s for each category); the variable measured is the number of correct items. To assess *inhibition*, we used the Stroop test [30], which consists of three successive tasks of 45 s each: word reading (W), color patch naming (C), and interference condition, which requires the participants to name the ink color and ignore the word (CW); the interference score that reflects inhibition abilities was calculated using the following formula: CW/[(W + C)/2], where CW, W, and C are the number of correct items in the corresponding task [31].

Finally, to assess *planning abilities*, we analyzed the strategies used by the subject when copying the complex Rey figure; the scores ranged from 1 to 4 (1 reflecting a perfect strategy).

*Functional mobility* was assessed using the timed up and go (TUG) test [32], which requires the participant to stand up from an armchair, walk 3 m, turn, walk back, and sit down, all at a comfortable pace. Two trials were given, and the best performance (shortest time) was recorded. The TUG test was not administered to fallers with lower limb fracture to avoid the subsequent effects of this fracture on gait.

#### Other outcomes

During the medical examination, a physician carefully collected information about drugs taken by participants via medical prescriptions, which was confirmed by a medication history interview. The physician also collected information on comorbidities (from the Kaplan–Feinstein scale: hypertension, cardiac, cerebral or psychic, respiratory, renal, hepatic, gastrointestinal, peripheral vascular, cancer, locomotor, alcoholism, and miscellaneous) [33], the number of previous falls experienced in the preceding twelve months, and risk factors for falls (hypotension, rheumatological disorders, tinnitus, muscular weakness, abnormal proprioceptive sensitivity in the lower limbs, and age ≥ 75 years). These risk factors were defined by consensus between physician-investigators after elimination of the exclusion criteria (e.g., low visual acuity, depression) and the factors used as variables of interest (BMD, cognition and mobility). Muscular strength was assessed with a handgrip dynamometer, which has been recognized as a good simple measure of muscle strength and correlates with leg strength [34]; each participant performed 2 trials per hand, and the best performance was recorded.

### Statistical analysis

We compared sociodemographic characteristics and outcome measures based on fall severity and bone density using the following tests: Student’s t test, the Mann‒Whitney test, the chi‒squared test, the Kruskal‒Wallis test, and ANOVA with the Bonferroni post hoc correction applied using the sequential method after pairwise comparisons. The normality of data distribution was verified by the Shapiro‒Wilk test. Cognitive and mobility data included both continuous raw scores and categorical impaired scores (MoCA and IST < 25), impaired scores for MMSE and TMT-A and TMT-B completion times range from < 23 to < 26, <62.60 to < 39 s, and < 157.95 to < 123.51 s, respectively, depending on educational level in persons aged 50 years and over, [35, 36] and impaired scores for the TUG range from ≥ 9 s to ≥ 12.7 s, depending on age [37].

We performed logistic regressions to test the association between variables and fracture occurrence using the backward stepwise method. Both univariate and multivariate regressions were performed. Co-variables included in the multivariate analysis were those that scored a p-value < 10% in the univariate analysis. Variables with ceiling or floor effects or which overlap were excluded. We also conducted a PCA with varimax rotation to identify key outcome measures explaining fracture variance. The Kaiser‒Meyer‒Olkin (KMO) measure ensured sampling adequacy. Components with eigenvalues > 1 and variables with loadings > 0.50 were retained [38]. To reduce any potential bias resulting from an expected strong association between women and fractures, the above analyses were also conducted separately for men and women.

All analyses were performed with SPSS 24.0^®^ software (IBM; Armonk, NY, USA), and the significance threshold was set at 0.05.

## Results

Out of the 376 injured fallers that agreed to participate in the study, 250 did not meet the inclusion criteria, resulting in 126 injured fallers included in the present study. After searching for non-injured fallers with a ratio of one-control for two cases, 63 individuals were added which brings the total to 189 participants. Fallers with a lower limb fracture represented 40% of the injured fallers. As shown in Table 1, the mean age of the study population was 71 years, and the majority were women (83%). The mean BMD T-score (-1.71 ± 3.75; -1.71 ± 1.02 for women and − 1.73 ± 0.86 for men) was within the osteopenic range. Global cognitive efficiency and mobility scores were within normal ranges (i.e., 27, 26 and 9.5 s for the MMSE, MoCA and TUG, respectively).

**Table 1:** Characteristics of the study population and comparisons between fall severity (with or without fracture) and bone density status

	ALL *N*=189	Fall- severity			Bone density status			
		Non-injured (*n*=63)	With fracture (*n*=126)	*p*-value	Normal BMD (*n*=73)	Osteopenia (*n*=78)	Osteoporosis (*n*=38)	*p*-value
**General characteristics**								
Age, years	71.05 ± 9.23	71.03 ± 9.71	71.08 ± 8.27	0.973^a^	67.55 ± 6.81	71.12 ± 10.23^$^	76.71 ± 8.64 ^*, #^	**0.001** ^**e**^
Education, years	11.34 ± 3.48	13.62 ± 4.57	11.97 ± 5.40	**0.001** ^**b**^	12.38 ± 4.07	12.21 ± 4.34	11.37 ± 3.31	0.653^d^
Women, number (%)	156(83)	38(63)	118(94)	**0.001** ^**c**^	61(83)	60(38)	33(88)	0.724^c^
Handgrip strength, kg	21.09 ± 7.93	24.70 ± 9.22	19.27 ± 6.52	**0.001** ^**a**^	21.95 ± 8.14	21.64 ± 8.20	18.60 ± 6.90	0.254^e^
BMI, kg/m^2^	27.33 ± 7.05	26.86 ± 5.15	29.95 ± 6.71	0.545^b^	29.32 ± 5.22	26.43 ± 4.56^$^	23.91 ± 4.16 ^*,#^	**0.001** ^**d**^
Comorbidities, number	1.76 ± 1.42	1.97 ± 1.53	1.66 ± 1.36	0.213^b^	1.79 ± 1.44	1.53 ± 1.50	2.0 ± 1.13	0.232^e^
Risk factors for falls, number	0.94 ± 0.95	0.78 ± 0.62	1.02 ± 0.98	0.076^a^	0.63 ± 0.48	0.95 ± 0.89^$^	1.45 ± 1.05 ^*,#^	**0.001** ^**e**^
Falls in past 12 months, number	1.85 ± 0.18	1.75 ± 0.20	1.89 ± 0.24	0.702^a^	1.95 ± 0.34	1.77 ± 0.25	1.84 ± 0.34	0.908^a^
Prescribed drugs, number	4.13 ± 3.75	5.30 ± 3.63	5.46 ± 4.13	0.786^c^	5.04 ± 3.58	5.20 ± 3.87	6.52 ± 4.78	0.153^e^
**Post-fall and bone density status**								
Fall-related fractures, number (%)	126(67)	0	100	NA	41(56)	47(60)	34(89) ^*, #^	**0.002** ^**c**^
BMD, T-score	-1.71 ± 3.75	-1.44 ± 0.89	-1.86 ± 1.02	**0.007** ^**a**^	-1.01 ± 0.77	-1.71 ± 0.51^$^	-3.08 ± 0.57 ^*, #^	**0.0001** ^**e**^
**Global cognition**								
MMSE score	27.59 ± 2.81	28.30 ± 1.69	27.73 ± 3.18	**0.028** ^**b**^	28.25 ± 1.92	27.82 ± 2.03	25.84 ± 4.57^*^	**0.004** ^**d**^
Impaired MMSE, number (%)	18(10)	5(8)	13(10)	**0.046** ^**c**^	4(5)	7(9)	7(18)	0.076^c^
MoCA score	26.01 ± 4.04	27.97 ± 2.52	25.91 ± 4.16	**0.001** ^**b**^	26.86 ± 3.50	26.48 ± 3.18	24.00 ± 5.46^*^	**0.005** ^**d**^
Impaired MoCA, number (%)	48(26)	6(10)	42(33)	**0.002** ^**c**^	13(18)	18(23)	17(45) ^*^	**0.035** ^**c**^
**Processing speed**								
TMT A score (sec)	40.08 ± 19.51	34.92 ± 11.02	42.73 ± 22.25	**0.041** ^**b**^	35.51 ± 12.9	38.47 ± 15.7	51.95 ± 30.8 ^*, #^	**0.001** ^**d**^
Impaired TMT A, number (%)	37(20)	9(14)	28(23)	0.118^c^	13(18)	14(19)	10(27)	0.527^c^
Zazzo, completion time (sec)	136.30 ± 47.7	126.5 ± 35.7	141.3 ± 52.3	0.135^b^	123.5 ± 37.3	131.0 ± 40.0	170.80 ± 65.0 ^*, #^	**0.001** ^**d**^
Rey Figure, copy time (sec)	178.1 ± 90.8	156.1 ± 84.11	200.6 ± 29.4	**0.004** ^**a**^	168.2 ± 86.2	170.8 ± 76.7	245.9 ± 99.6 ^*, #^	**0.006** ^**d**^
**Memory**								
Forward digit span (score)	7.73 ± 1.94	8.16 ± 1.87	7.52 ± 1.95	**0.033** ^**a**^	8.0 ± 1.92	7.88 ± 2.0	7.11 ± 1.85	0.065^e^
Backward digit span (score)	5.30 ± 1.52	5.70 ± 1.73	4.91 ± 1.75	**0.004** ^**a**^	5.45 ± 2.04	5.32 ± 1.62	4.43 ± 1.36^*, #^	**0.013** ^**e**^
Letter digit sequence (score)	8.67 ± 2.85	9.24 ± 2.96	8.36 ± 2.75	**0.042** ^**a**^	8.99 ± 2.61	9.10 ± 3.04	7.44 ± 2.57^*, #^	**0.029** ^**e**^
Rey Figure, recall (score)	16.30 ± 4.77	15.54 ± 5.78	15.98 ± 5.22	0.135^b^	15.82 ± 5.68	14.60 ± 6.10	13.62 ± 5.94	0.132^d^
**Flexibility and Fluency**								
TMT-B completion time (sec)	97.66 ± 61.80	83.29 ± 35.75	105.29 ± 70.9	**0.027** ^**b**^	86.37 ± 47.55	95.85 ± 49.71	123.14 ± 97.44^*^	0.077^d^
Impaired TMT-B, number (%)	31(16)	8(13)	23(20)	0.175^c^	12(17)	17(22) ^$^	2(4)	**0.039** ^**c**^
TMT B-A score (sec)	59.66 ± 51.08	49.24 ± 30.70	65.19 ± 58.51	**0.047** ^**b**^	51.40 ± 40.19	57.90 ± 39.51	78.28 ± 81.23^*^	**0.028** ^**d**^
Impaired TMT B-A, number (%)	22(12)	5(8)	17(15)	0.153^c^	7(10)	8(12)	7(17)	0.095^c^
Isaacs Set test (IST) score	36.22 ± 7.42	38.19 ± 7.15	35.22 ± 7.38	**0.009** ^**a**^	38.04 ± 7.18	36.61 ± 6.55	32.30 ± 8.22^*, #^	**0.001** ^**e**^
Impaired IST, number (%)	43(23)	11 (18)	32(26)	0.136^c^	18(25)	15(19)	10(26)	0.625^c^
**Inhibition**								
Stroop (score)	0.48 ± 0.10	0.47 ± 0.08	0.51 ± 0.14	0.055^a^	0.50 ± 0.11	0.47 ± 0.10	0.49 ± 0.09	0.077^e^
**Planning abilities**								
Rey Figure, copying strategy (score)	2.32 ± 1.15	2.16 ± 1.24	2.48 ± 1.32	**0.042** ^**a**^	2.47 ± 1.35	2.24 ± 1.27	2.51 ± 1.30	0.490^d^
**Functional mobility**								
TUG^€^ (sec)	9.50 ± 3.48	8.63 ± 1.92	10.24 ± 4.29	**0.005** ^**a**^	9.11 ± 2.54	8.75 ± 1.95	12.64 ± 6.74^*,#^	**0.001** ^**e**^
Impaired TUG, number (%)	44(24)	17(27)	27(37)	**0.001** ^**c**^	24(33)	13(16) ^$^	7(18)	**0.001** ^**c**^

Unless indicated, values are mean ± SD; ^a^Student’s t-test, ^b^U test of Mann-Whitney, ^c^Chi-square test, ^d^Kruskall-Walis or ^e^One-way ANOVA. BMI: Body Mass Index; BMD: Bone Mineral Density; MMSE: Mini Mental State Examination; MoCA: Montreal Cognitive Assessment; IST: Isaacs Set Test; TUG: Time Up and Go; TMT: Trail Making Test. Impaired scores for MoCA and Isaacs Set test are < 25. Impaired scores for the MMSE, TMT-A and TMT-B completion time range from <23 to <26, <62.60 to <39 s, and <157.95 to <123.51 s, respectively, depending on educational level in persons aged 50 years and over. Impaired scores for the TUG range from ≥9 s to ≥12.7 s, depending on age. Significant post-hoc intergroup differences are represented by the following symbols: $ for or “osteopenia vs. normal BMD”, * for “osteoporosis vs. normal”, and # for “osteoporosis vs. osteopenia”; the p-values of the post-hoc analyses are specified in the text. ^€^*n*=123 participants only because not tested in fallers with lower limb fracture so as to avoid this fracture’s after effects on gait

Intergroup comparisons revealed that fallers with fractures were significantly less educated, more often women, and had lower handgrip strength and T scores than non-injured fallers (*p* ≈ 0.001 each). They also had poorer scores in global cognition (MMSE *p* = 0.028, MoCA *p* = 0.001), processing speed (TMT-A *p* = 0.041, Rey Figure-copy time *p* = 0.004), memory (*p* = 0.037, 0.004 and 0.044 for the forward and backward digit span, and the letter digit sequence, respectively), mobility (TUG *p* = 0.005) and others (see Table 1). Additionally, they had greater proportions of impaired MMSE, MoCA, and TUG scores than did the non-injured group (*p* = 0.042, 0.002, 0.001, respectively).

Significant differences in general characteristics and cognitive and mobility scores were found when comparing participants by bone density status (Table 1). Bonferroni post hoc analyses showed that, compared to both the normal BMD and osteopenia groups, those with osteoporosis were older (*p* = 0.001, 0.005), had a lower BMI (*p* = 0.001, 0.027), had more fall risk factors (*p* = 0.001, 0.019), and had a greater proportion of fall-related fractures. The participants also had poorer scores for global cognition (MMSE: *p* = 0.001, 0.028; MoCA: *p* = 0.001, 0.002), processing speed (TMT A: *p* = 0.003, 0.025; Rey Figure-copy time: *p* = 0.001, 0.011; Zazzo completion time: *p* = 0.001, 0.002), working memory (backward digit span: *p* = 0.001, 0.002; letter digit sequence: *p* = 0.029, 0.017), flexibility, fluency (TMT B-A vs. normal BMD: *p* = 0.039; Isaacs Set Test: *p* = 0.003, 0.045), and mobility (TUG: *p* = 0.015, 0.003). The osteoporosis group had more impaired MoCA scores than did the normal BMD group (*p* = 0.025). Additionally, the osteopenia group was older (*p* = 0.041) and had a lower BMI (*p* = 0.001), more fall risk factors (*p* = 0.043), and more impaired performance on the TMT-B (*p* = 0.038) and TUG (*p* = 0.011) tests than the normal BMD group.

The following variables, which showed ceiling or floor effects in their distribution or were considered overlapping variables, were then excluded from the regression analysis: MMSE, MoCA, Rey Figure (copy time, recall and strategy), forward digit span, TMT-B completion time, and TUG. As shown in Table 2, the results of the multivariate regression computed across all participants revealed an independent association between the presence of fracture and the following variables: age (OR = 0.93, 95% CI = 0.88–0.98, *p* = 0.010), education (OR = 0.87, 95% CI = 0.79–0.97, *p* = 0.013), BMD (OR = 0.49, 95% CI = 0.30–0.81, *p* = 0.015), percentage of women (OR = 8.42, 95% CI = 3.15–22.51, *p* = 0.00002), and TMT-A score (OR = 1.03, 95% CI = 1.01–1.08, *p* = 0.034).

**Table 2:** Results from the logistic regressions performed to analyze the relationships between fall severity (absence vs. presence of fracture) and variables across all participants

	Univariate			Multivariate		
	OR	95% CI	*p*-value	OR	95% CI	*p*-value
Age	0.97	0.93-1.02	**0.079**	0.93	0.88-0.98	**0.013**
Education	0.89	0.80-0.90	**0.042**	0.87	0.79-0.97	**0.013**
Percentage of women	9.70	4.04-23.30	**0.0001**	8.42	3.15-22.51	**0.00002**
BMI	1.04	0.96-1.13	0.286			
BMD	0.54	0.36-0.81	**0.003**	0.47	0.29-0.75	**0.001**
Handgrip strength	0.97	0.92-1.03	0.359			
Zazzo completion time	0.99	0.97-1.01	0.293			
TMT-A completion time	1.03	1.01-1.05	**0.010**	1.03	1.01-1.08	**0.034**
TMT B-A	1.03	0.98-1.04	0.706			
IST score	0.99	0.91-1.07	0.804			
Backward digit span score	0.86	0.74-0.99	**0.040**			
Letter digit sequence score	0.91	0.74-1.11	0.372			
Stroop Score	0.11	0.01-14.79	0.387			
Risk factors for falls, number	1.15	0.73-1.82	0.539			
Comorbidities, number	0.96	0.72-1.28	0.814			
Prescribed drugs, number	1.13	0.47-2.68	0.775			

BMI: Body Mass Index; BMD: Bone Mineral Density; TMT: Trail Making Test; IST: Isaacs Set Test

Regarding the sex-specific analyses, as displayed in Table 3, the logistic regressions conducted in women showed that the presence of fracture was independently associated with age (OR = 0.90, 95% CI = 0.84–0.95, *p* = 0.001), BMD (OR = 0.43, 95% CI = 0.25–0.75, *p* = 0.001), backward digit span score (OR = 0.77, 95% CI = 0.60–0.98, *p* = 0.039), and TMT-A score (OR = 1.05, 95% CI = 1.01–1.10, *p* = 0.017). In male participants, logistic regressions did not reveal any associations between the presence of a fall-related fracture and the variables of interest (data not shown).

**Table 3:** Results from the logistic regressions performed to analyze the relationships between fall severity (absence vs. presence of fracture) and variables in women

	Univariate			Multivariate		
	OR	95% CI	*p*-value	OR	95% CI	*p*-value
Age	0.99	0.95-1.03	**0.087**	0.90	0.84-0.95	**0.001**
Education	0.88	0.79-0.97	**0.015**			
BMI	0.98	0.92-1.05	0.729			
BMD	0.55	0.37-0.82	**0.003**	0.43	0.25-0.75	**0.003**
Handgrip strength	0.97	0.90-1.03	0.336			
Zazzo completion time	1.01	0.97-1.02	0.186			
TMT-A completion time	1.03	1.01-1.07	**0.022**	1.05	1.01-1.10	**0.017**
TMT B-A	1.01	0.99-1.02	**0.069**			
IST score	0.92	0.88-0.97	**0.006**			
Backward digit span score	0.75	0.61-0.93	**0.010**	0.77	0.60-0.98	**0.039**
Letter digit sequence score	0.86	0.75-1.00	**0.051**			
Stroop Score	0.04	0.01-2.63	0.135			
Risk factors for fall, number	1.09	0.74-1.60	0.639			
Comorbidities, number	0.87	0.75-1.27	0.876			
Prescribed drugs, number	1.07	0.51-2.24	0.850			

BMI: Body Mass Index; BMD: Bone Mineral Density; TMT: Trail Making Test; IST: Isaacs Set Test

The results from the PCA across all 189 participants are shown in Table 4. The KMO measure (0.749) indicated that the sample size was adequate. We identified four components, C1 to C4, with eigenvalues > 1; these four components explained 67% of the total variance. The distribution of the variables within the components was as follows: C1 (age, Zazzo and TMT-A completion times, TMT B-A, and risk factor for falls), C2 (education, backward digit span and letter digit sequence scores), C3 (percentage of women and handgrip strength) and C4 (BMD, BMI). The results of the PCA performed for women (KMO = 0.796) are shown in Table 5. Three independent components, C1_w_ to C3_w_, explained 54% of the variance. The variable distribution was very similar to that across all participants, although it was grouped into only 3 components, with the first one including more cognitive variables than in all participants: C1_w_ (Zazzo and TMT-A completion times, TMT B-A, IST, Stroop, backward digit span and letter digit sequence scores, and education), C2_w_ (age, handgrip strength, and risk factors for falls), and C3_w_ (BMD, BMI, and number of comorbidities).

**Table 4:** Components matrix after varimax rotation, across all participants

	Components			
	C1	C2	C3	C4
Age	0.746			
Zazzo completion time	0.715			
TMT-A completion time	0.614			
TMT B-A	0.578			
Risk factors for falls, number	0.633			
BMI				0.866
BMD				0.741
Women (%)			0.900	
Handgrip strength (Kg)			0.851	
Backward digit span score		0.833		
Letter digit sequence score		0.772		
Education (years)		0.675		
Eigenvalues	4.571	2.106	1.712	1.250
Total variance (%)	28.57	13.16	10.70	7.81

BMI: Body Mass Index; BMD: Bone Mineral Density; TMT: Trail Making Test

**Table 5:** Components matrix after varimax rotation in women

	Components		
	C1_w_	C2 _w_	C3 _w_
Zazzo completion time	-0.609		
TMT-A completion time	-0.703		
TMT B-A	-0.635		
IST score	0.740		
Stroop score	0.534		
Backward digit span score	0.657		
Education (years)	0.532		
Letter digit sequence score	0.760		
BMI			0.638
BMD			0.820
Age		0.774	
Handgrip strength (Kg)		-0.694	
Risk factors for falls, number		0.709	
Comorbidities, number			0.638
Eigenvalues	4.676	2.098	1.392
Total variance (%)	31.17	13.98	9.20

BMI: Body Mass Index; BMD: Bone Mineral Density; TMT: Trail Making Test; IST: Isaacs Set Test

Due to the lack of significant findings from the logistic regression in men, PCA was not performed for this subgroup.

## Discussion

This is the first study to our knowledge to compare the involvement of cognitive abilities and bone density in fractures among community-dwelling fallers aged 55 years and older. Our findings indicate poorer cognitive performance in fractured participants than in non-injured fallers and also among osteoporotic fallers than in those with osteopenia and normal BMD. Logistic regression analysis indicated that poor cognitive abilities, particularly processing speed and working memory, were independent of BMD and other fracture risk factors in fallers with fractures, predominantly among women.

There were numerous differences when considering fall severity, as well as between participants with and without osteoporosis. In agreement with the literature, fallers who experienced a fracture were mainly women [3], had poorer muscle strength and were less educated than non-injured fallers. Furthermore, the osteoporotic group had a lower BMI and more risk factors for falls than the other two groups, as also reported before [3]. Interestingly, we also found that fallers who experienced a fracture and those with osteoporosis had poorer cognitive and mobility performance than non-injured fallers and those with normal BMD or osteopenia, respectively. This could suggest a link between osteoporosis, fracture and cognitive abilities. The logistic regression and PCA analyses further specify the nature of this link.

The poor cognitive abilities of fallers who experienced a fracture is in line with previous data that have clearly established a strong link between cognition, falls and fractures in both cross-sectional and prospective studies, with a high risk of fracture in people with poor cognition [11–15, 17]. While our study confirmed the involvement of poor cognitive abilities in fractured fallers, it revealed the most influential cognitive functions, which could not be identified in previous studies that focused mainly on global cognition and sometimes executive function. Our comprehensive cognitive assessment revealed significantly lower performance in processing speed, memory (particularly working memory), mental flexibility, verbal fluency, and planning among fractured versus non-injured fallers, with inhibition showing a trend toward significance. Notably, most participants showed cognitive performance within the normal range. Only 10 and 8% of the injured and non-injured fallers, respectively, had impaired MMSE scores, and the mean scores were relatively high (21.35 and 23.5, respectively). This suggests that the risk of fracture may manifest as soon as cognitive performance becomes nonoptimal.

The cross-sectional association between fractures in fallers and poor cognitive abilities, irrespective of BMD, is a novel finding. Multivariate regression revealed that poor processing speed was the main cognitive variable linked to fractures in fallers, independent of BMD. As individuals age, processing speed diminishes, impacting various cognitive domains and increasing fracture risk regardless of bone fragility. Notably, most people who fracture after a fall have a normal BMD [5, 9, 39]. In addition, this distinct involvement of cognitive abilities and osteoporosis in fracture occurrence is in line with our recent study, which identified different profiles in older women who experienced a fracture of the upper or lower limb following a fall [17]. Indeed, among the 4 identified profiles, two were clearly opposed in terms of osteoporosis and cognitive performance: the profile with the most osteoporotic participants (30%) had the best cognitive performance, whereas one of the two profiles with no osteoporotic participants displayed the lowest cognitive performance. It could thus be assumed that in the case of normal BMD, poor cognition could have significantly contributed to the fracture, at least in some of them.

Interestingly, the low cognitive performance in case of fracture occurrence in fallers was replicated in the gender-specific analyses performed among women, highlighting the involvement of processing speed and working memory. This finding is all the more relevant as the PCA in women identified TMT-A and backward digit span in the variables of the C1_w_ component, which explained the most variance (31%). The fact that the involvement of cognition in fracture was identified for working memory but not for executive functions might seem odd at first sight. Indeed, few studies have identified the contribution of poor executive functioning to fracture occurrence [11, 14, 15]. Nevertheless, it is well known that poor performance in attention, executive function, and working memory are associated with gait instability and that these cognitive disorders are predictive of falls [10]. Furthermore, reports have indicated that working memory demand affects accurate reactions following a loss of balance, especially in fallers [40]. In addition, working memory is classically recognized as being at the interface between memory, attention, and executive functions, and it is often considered that the central executive component of working memory is an executive function and that executive function includes working memory [41–43].

The absence of the polypharmacy variable in the 4 PCA profiles identified may seem surprising. It is well-established that polypharmacy, particularly involving psychotropic or anticholinergic drugs, significantly contributes to fracture occurrence. Since these drugs are also known to impact both cognitive abilities and mobility [44–46], future studies should investigate whether their contribution to fracture risk is mediated by their effects on cognitive functions.

Some limitations can be addressed. First, one could argue that the present findings may not be relevant to the general population since the participants were exclusively fallers. Nevertheless, as previously discussed in detail [45], our population is very similar to the general population, as the mean cognitive and mobility scores fall within the normal ranges. In addition, because 30% of individuals older than 65 years fall every year [2] and since we specifically searched for individuals who had fallen in the previous year, after our 5 years of inclusion, we likely collected a significant part of the general population. Second, significant relationships between the presence of a fall-related fracture and poor performance of specific cognitive functions in women were not found in men. Nevertheless, we found similar results across all participants and among women. In addition, given the absence of notable discrepancies between men and women across all variables of interest (see supplementary Table S1), it is likely that the absence of a relationship between fracture and cognition might result from the relatively small sample of men. The fact that only 20% of men were recruited could be explained by various factors, such as the higher prevalence of osteoporosis in women than in men with advancing age and the lower participation rate of men in research investigations addressing this topic [15, 17]. However, further studies are required to test this relationship in a larger sample of men. Assessment of bone microarchitecture, using TBS for instance, could also have provided interesting complementary information to BMD. However, it has been shown in population-based studies that TBS has the same discriminatory value, and no additive value, for fracture discrimination as compared to BMD alone [47]. Furthermore, the absence of a follow-up study confirming the relationships between specific cognitive abilities and fracture occurrence in a longitudinal setting could also be a limitation. However, the cross-sectional association revealed in this study aligns with the increasing body of evidence highlighting the influence of cognition on the occurrence of fractures in fallers.

## Conclusion

This study highlights the involvement of poor cognitive abilities in fractures among community-dwelling fallers aged 55 and over. Cognitive impairments were greater in fallers with fractures and in participants with osteoporosis than in those with osteopenia or normal BMD. Notably, poor cognitive abilities, particularly processing speed and working memory, in case of fracture occurrence was independent of bone fragility, especially in women. These findings underscore the importance of assessing cognitive abilities in managing fractures. Recent recommendations have urged the inclusion of routine cognitive assessments in multifactorial fracture risk assessments in older adults [2]. We recommend extending cognitive assessments to younger fallers (aged 55 years), especially those with osteoporosis, comorbidities, and other common fracture risk factors. This will ensure the best possible prevention.

## Electronic supplementary material

Below is the link to the electronic supplementary material.

Supplementary Material 1

## Data Availability

The data that support the findings of this study are available on request to the corresponding author. The data are not publicly available due to privacy or ethical restrictions.
