# Supplementary material for: Involvement of cognitive abilities in the occurrence of fractures in fallers aged 55 years or older: a cross-sectional study
Source: Aging Clin Exp Res. 2024 Aug 30;36(1):180. doi: 10.1007/s40520-024-02830-7 (PMC11364792; doi:10.1007/s40520-024-02830-7)
Supplement: Supplementary file 1 — Supplementary Material 1 [file 40520_2024_2830_MOESM1_ESM.docx]

**Supplementary Table S1.** Characteristics of the study population and comparison between men and women

|  | ALL  N=189 |  | **Gender comparisons** | | |
| --- | --- | --- | --- | --- | --- |
|  |  |  | Men  (n=33) | Women  (n=156) | *p*-value |
| Age, years | 71.05 ± 9.23 |  | 70.58 ± 8.51 | 71.15 ± 9.40 | 0.748^a^ |
| Education, years | 11.34 ± 3.48 |  | 13.73 ± 5.34 | 11.76 ± 3.59 | 0.057^b^ |
| Handgrip strength, kg | 21.09 ± 7.93 |  | 32.42 ± 8.17 | 18.63 ± 5.33 | **0.000001^a^** |
| BMI, kg/m^2^ | 27.33 ± 7.05 |  | 27.74 ± 4.00 | 26.86 ± 5.31 | 0.366^b^ |
| Comorbidities, number | 1.76 ± 1.42 |  | 2.27 ± 1.52 | 1.98 ± 1.38 | 0.062^b^ |
| Risk factors for falls, number | 0.94 ± 0.95 |  | 0.61 ± 0.60 | 1.01 ± 0.99 | **0.026^a^** |
| Falls in past 12 months, number | 1.85 ± 0.18 |  | 1.94 ± 0.20 | 1.36 ± 0.16 | **0.030^a^** |
| Prescribed drugs, number | 4.13 ± 3.75 |  | 5.30 ± 3.63 | 5.46 ± 4.13 | 0.786^c^ |
| Fall-related fractures, number (%) | 126(67) |  | 8(24) | 118(75) | **0.00000001**^c^ |
| BMD, T-score | -1.71 ± 3.75 |  | -1.73 ± 0.86 | -1.71 ± 1.02 | 0.912^a^ |
| MMSE score | 27.59 ± 2.81 |  | 28.03 ± 1.96 | 27.49 ± 2.96 | 0.028^b^ |
| Impaired MMSE, number (%) | 18(10) |  | 3(10) | 15(9) | 0.613^c^ |
| MoCA score | 26.01 ± 4.04 |  | 26.79 ± 3.56 | 25.85 ± 4.13 | 0.157^b^ |
| Impaired MoCA, number (%) | 48(26) |  | 7(21) | 41(26) | 0.357^c^ |
| TMT A score (sec) | 40.08 ± 19.51 |  | 34.92 ± 11.02 | 42.73 ± 22.25 | 0.873^b^ |
| Impaired TMT A, number (%) | 37(20) |  | 32(21) | 5(15) | 0.313^c^ |
| Zazzo, completion time (sec) | 136.30 ± 47.7 |  | 131.6 ± 48.5 | 133.2 ± 47.7 | 0.135^b^ |
| Rey Figure, copy time (sec) | 178.1 ± 90.8 |  | 153.8 ± 72.40 | 192.4 ± 24.5 | 0.082^a^ |
| Forward digit span (score) | 7.73 ± 1.94 |  | 8.09 ± 1.80 | 7.66 ± 1.97 | 0.435^a^ |
| Backward digit span (score) | 5.30 ± 1.52 |  | 5.30 ± 1.86 | 5.15 ± 1.77 | 0.154^a^ |
| Letter digit sequence (score) | 8.67 ± 2.85 |  | 8.79 ± 3.36 | 8.64 ± 2.73 | 0.148^a^ |
| Rey Figure, recall (score) | 16.30 ± 4.77 |  | 16.13 ± 6.8 | 14.45 ± 5.7 | 0.135^b^ |
| TMT-B completion time (sec) | 97.66 ± 61.80 |  | 98.81 ± 50.3 | 97.40 ± 64.2 | 0.619^b^ |
| Impaired TMT-B, number (%) | 31(16) |  | 8(10) | 23(20) | 0.143^c^ |
| TMT B-A score (sec) | 59.66 ± 51.08 |  | 61.75 ± 42.23 | 59.18 ± 53.01 | 0.798^b^ |
| Impaired TMT B-A, number (%) | 22(12) |  | 4(13) | 18(13) | 0.585^c^ |
| IST score | 36.22 ± 7.42 |  | 35.76 ± 6.13 | 36.32 ± 7.68 | 0.695^a^ |
| Impaired IST, number (%) | 43(23) |  | 4(12) | 39(25) | 0.075^c^ |
| Stroop score | 0.48 ± 0.10 |  | 0.50 ± 0.14 | 0.48 ± 0.09 | 0.231^a^ |
| Rey Figure, copying strategy (score) | 2.32 ± 1.15 |  | 2.06 ± 1.24 | 2.44 ± 1.30 | 0.249^a^ |
| TUG^€^ (sec) | 9.50 ± 3.48 |  | 9.01 ± 2.01 | 9.63 ± 3.80 | 0.388^a^ |
| Impaired TUG, number (%) | 44(24) |  | 10(30) | 34(22) | **0.014^c^** |

Unless indicated, values are mean ± SD; ^a^Student’s t-test, ^b^U test of Mann-Whitney, ^c^Chi-square test. BMI: Body Mass Index; BMD: Bone Mineral Density; MMSE: Mini Mental State Examination; MoCA: Montreal Cognitive Assessment; IST: Isaacs Set Test; TUG: Time Up and Go; TMT: Trail Making Test. Impaired scores for MoCA and Isaacs Set test are < 25. Impaired scores for the MMSE, TMT-A and TMT-B completion time range from <23 to <26, <62.60 to <39 sec, and <157.95 to <123.51 sec, respectively, depending on educational level in persons aged 50 years and over. Impaired scores for the TUG range from ≥9 sec to ≥12.7 sec, depending on age. ^€^n=123 participants only because not tested in fallers with lower limb fracture so as to avoid this fracture’s after effects on gait.
